# The Impact of SARS-CoV-2 Infection, and Application of Immunosuppressive Agents in Kidney Transplant Recipients Suffering from COVID-19

**DOI:** 10.3390/ph14101054

**Published:** 2021-10-17

**Authors:** Horng-Ta Tseng, Xiang-Chi Wu, Chun-Yao Huang, Chun-Ming Shih, Yi-Wen Lin, Feng-Yen Lin

**Affiliations:** 1 Taipei Heart Institute, Taipei Medical University, Taipei 110, Taiwan; b101107097@tmu.edu.tw (H.-T.T.); b101107042@tmu.edu.tw (X.-C.W.); cyhuang@tmu.edu.tw (C.-Y.H.); cmshih53@tmu.edu.tw (C.-M.S.); 2 Division of Cardiology and Cardiovascular Research Center, Taipei Medical University Hospital, Taipei 110, Taiwan; 3 Departments of Internal Medicine, School of Medicine, College of Medicine, Taipei Medical University, Taipei 110, Taiwan; 4 Institute of Oral Biology, National Yang Ming Chiao Tung University, Taipei 112, Taiwan

**Keywords:** COVID-19, SARS-CoV-2, solid organ transplant, kidney transplantation, immunosuppressive agents

## Abstract

In December 2019, the COVID-19 pandemic began to ravage the world quickly, causing unprecedented losses in human life and the economy. A statistical study revealed that the proportion of solid organ transplant (SOT) recipients with severe symptoms and deaths after being infected by SARS-CoV-2 is considerably higher than that of non-SOT recipients, and the prognosis is relatively poor. In addition, the clinical manifestation of SOT recipients suffering from COVID-19 is different from that of general COVID-19 patients. Acute kidney injury (AKI) is a common complication in COVID-19 patients, and it is likely more common among SOT recipients infected with SARS-CoV-2. Clinical experts consider that SOT recipients have long-term treatment with immunosuppressants, and the comorbidities are driven by a high rate of severe symptoms and mortality. Orthotopic kidney allograft transplantation is an effective treatment for patients suffering from end-stage kidney disease/kidney failure through which they can easily extend their life. Indeed, kidney transplant recipients have suffered significant damage during this pandemic. To effectively reduce the severity of symptoms and mortality of kidney transplant recipients suffering from COVID-19, precise application of various drugs, particularly immunosuppressants, is necessary. Therefore, herein, we will collate the current clinical experience of treating COVID-19 infection in kidney transplant recipients and discuss the adjustment of patients using immunosuppressive agents in the face of COVID-19.

## 1. Introduction

December 2019 marked the outbreak of severe acute respiratory syndrome worldwide. Scientists discovered that a virus, coronavirus-2 (SARS-CoV-2), was the major cause of the disease. Subsequently, the World Health Organization (WHO) defined it as coronavirus disease 2019 (COVID-19). Since the outbreak of the pandemic, COVID-19 has rapidly ravaged the world, causing the loss of a large number of lives and huge economic losses [1]. Kidney transplant patients have also been severely impacted during this pandemic. Orthotopic kidney allograft transplantation is an effective treatment for extending the life of patients suffering from end-stage kidney disease or kidney failure. To control the immune response and rejection that occurs in solid organ transplant (SOT) recipients and to increase the life span of the donor graft, patients receive immunosuppressive medications [2]. However, when these patients suffer from COVID-19, the use of immunosuppressive agents and the comorbidities of the patients may become factors affecting their ability to fight against SARS-CoV-2 infection and recover [3]. The effects of immunosuppressive agents, including immunosuppression, co-infection, and drug-induced leukopenia, may be related to the severity of the patient’s COVID-19 or their mortality [4,5]. SOT recipients have a higher chance of developing severe illness and death than others after suffering from COVID-19infection, and the prognosis is worse than that of ordinary people [6,7]. Several studies have also proposed opinions regarding the immune response caused by SARS-CoV-2 infection. The research reports indicate that the clinical manifestations of SOT recipients with COVID-19 are different from those of non-SOT COVID-19 patients. SOT recipients infected with COVID-19 have lower rates of fever, more significant lymphopenia, higher rates of gastrointestinal problems, and higher fatality rates compared to non-SOT COVID-19 patients [8,9,10,11]. Acute kidney injury (AKI) is a common complication in COVID-19 patients, and it appears to have a higher incidence in kidney transplant recipients infected with COVID-19 [3,12]. However, renal abnormalities may cause these SOT recipients to have a high mortality rate [12]. In addition, patients in this category have higher rates of SARS-CoV-2 RNAemia and higher rates of co-infection than immune competent individuals [4,13]. Divergent research findings also exist; a cohort study reported that the intensity of immunosuppression, duration of immunosuppression treatment, number of immunosuppressive agents used, and type of organ transplantation do not have any influence on the mortality rate of patients infected with COVID-19. In contrast, age, chronic heart failure, chronic lung disease, obesity, lymphopenia, and radiographic abnormalities are associated with the mortality rate of COVID-19 patients [11]. In another study, it was also reported that the results of analysis after excluding the above mentioned factors that may be related to mortality (e.g., age, chronic heart failure, chronic lung disease, obesity, lymphopenia, and radiographic abnormalities), the clinical outcome of COVID-19 between SOT recipients and non-SOT patients is not significantly different [14,15]. Although some studies have found that SOT or immunosuppression caused by drugs after SOT may not be related to the mortality from COVID-19, most studies have shown that SOT recipients infected with SARS-CoV-2 tend to have more serious symptoms than those without SOT. Clarifying the complex interactions of SARS-CoV-2 with the host immune system and drugs can enable better understanding of COVID-19. Therefore, we collate the current clinical treatment experiences of kidney transplantation recipients with COVID-19 and discuss how clinicians use immunosuppressive or immunomodulatory agents for these patients in the face of COVID-19 treatment in this article. Through this paper, we aim to provide suggestions and ideas related to COVID-19 treatment for kidney transplantation recipients.

## 2. Impact of SARS-CoV-2 Infection on the Immune System of Patients

### 2.1. Impact on Non-SOT Patients

Coronaviruses cause human and animal diseases. Most coronaviruses only infect the upper respiratory tract, causing relatively mild symptoms. However, severe acute respiratory syndrome coronavirus-1 (SARS-CoV-1), Middle East respiratory syndrome coronavirus (MERS-CoV), and SARS-CoV-2 can infect the lower respiratory tract and cause severe pneumonia [1,16]. SARS-CoV-2 infection can result in severe respiratory symptoms. The clinical manifestations of COVID-19 include fever, fatigue, dry cough, dyspnea, and acute respiratory distress syndrome (ARDS) [17]. SARS-CoV-2 binds to cellular angiotensin-converting enzyme 2 (ACE2) receptors by the S protein on its surface, thereby entering and infecting airway epithelial cells [18]. The stimulation of SARS-CoV-2 causes Th1 cells to secrete pro-inflammatory cytokines, such as interleukin (IL)-6 and granulocyte-macrophage colony-stimulating factor (GM-CSF). SARS-CoV-2 can also trigger the infiltration of a large number of monocytes and macrophages into the lungs [19], and this rising number of monocytes and macrophages are related to the production of pro-inflammatory cytokines, such as IL-6, tumor necrosis factor-α (TNF-α), IL-1, and IL-8. These cytokines trigger violent immune responses in patients and induce cytokine storms. The COVID-19 course is divided into three stages. Stage I (early infection): at this stage, the virus proliferates in the cells, causing the cells to undergo morphological changes (called cytopathic effect). At this time, the patient will primarily show mild symptoms. Stage II (pulmonary phase): starting approximately 7–10 days after the infection of SARS-CoV-2, the virus will invade the lungs on a large scale (called progressive lung involvement), and the patient will develop pneumonia with cough and/or fever. The consumption of oxygen and demand for ventilatory support will increase at this stage. If the patient’s condition worsens, it will progress to Stage III (systemic hyper-inflammation). At this stage, the body will produce cytokine-release syndrome, also known as a cytokine storm, causing systemic hyper-inflammation. The symptoms of severe ARDS, systemic inflammatory response syndrome (SIRS), shock, and cardiac failure will appear. Patients at this stage typically require the assistance of mechanical ventilation or extracorporeal membrane oxygenation (ECMO) to support hemodynamic situation [20,21,22,23]. In the analysis report of transcriptional and serum profiling of COVID-19 patients, a special and unusual inflammatory response is presented, in which the response of type 1 interferons, which play an important role in antiviral immunity, is reduced. In addition, the inflammatory response induced by NF-κB is strengthened, and serum IL-6 and TNF-α have an upward trend [24,25]. Some laboratory data also revealed that C-reactive protein (CRP) and IL-6 in serum may be the factors that affect the severity of COVID-19. High levels of CRP and IL-6 have been identified as mortality predictors [26,27,28]. In addition, COVID-19 patients typically develop lymphopenia, and the severity and mortality of patients who develop lymphopenia will be higher than those without lymphopenia [29]. The reduction of CD3-, CD4-, and CD8-positive lymphocytes in the blood may be caused by lymphocyte exhaustion, virus-induced death of lymphocytes, pulmonary recruitment of lymphocytes, and cytokine storm. Immunosuppressive agents (including lymphocyte-depleting agents and anti-metabolites) may also cause the augmentation of lymphopenia in SOT recipients [17,30]. As T cells have a regulatory effect on the over-activated immune response [31], the increase in neutrophil-to-lymphocyte ratio (NLR) is typically accompanied by lymphopenia. The increase in NLR is related to the severity of the disease, and patients with COVID-19 show that the increase in NLR is more significant than that of general viral pneumonia patients, which means that the severity of COVID-19 is greater than that of general viral pneumonia. [32,33,34]. Regarding the humoral immune response, patients will produce anti-SARS-CoV-2-neutralizing antibodies after being infected with SARS-CoV to limit viral replication and inhibit disease progression. If the disease becomes serious, the humoral immune response will have a tendency to strengthen [35].

### 2.2. Impact on SOT Recipients

Previous studies have reported that, compared to non-SOT patients, SOT recipients have different clinical signs and outcomes after being infected with SARS-CoV-2, including a higher probability of gastrointestinal problems, such as diarrhea and nausea. AKI has also been found in hospitalized kidney transplant recipients with COVID-19 [3,12]. This phenomenon may be due to SOT recipients receiving immunosuppressive treatment [7,9,36]. As SOT induces chronic rejection and leads to allograft vasculopathy or vascular damage, vascular damage will also induce thrombotic complications and reduce renal perfusion, eventually leading to graft failure [12,37]. Therefore, when a kidney transplant patient has progressive allograft vasculopathy or vascular damage, SARS-CoV-2 infection can easily induce AKI and increase hospitalization mortality [3,12,38]. SOT recipients with COVID-19 are more likely to have lymphopenia and are more seriously infected than non-SOT patients with COVID-19 [4,39]. At present, there is not enough information about the humoral immune response in SOT recipients with COVID-19 and non-SOT patients with COVID-19. Compared with non-SOT patients, SOT recipients have higher rates of SARS-CoV-2 RNAemia. This may be due to the weaker or incomplete immunity of transplant recipients, which leads to a decrease in the clearance ability of the virus [4]. Co-infection is also common in COVID-19 patients, and co-infection is typically one of the factors in the death of COVID-19 patients. Statistical analysis shows that the co-infection rate that occurred in non-survivors of COVID-19 was 50% [40,41]. The co-infection rate of SOT recipients is higher than that of non-transplant patients, and it has a negative effect on their prognosis [42,43]. Some scholars have suggested evaluating the status of bacterial and opportunistic fungal infections in SOT recipients with COVID-19 [42,43] for reference in the treatment process and increase the recovery rate [42,43].

As for the intensity of suppressed immunity in patients, the risk and severity of SARS-CoV-2 infection have also been continuously discussed. Patients will be treated with a large number of immunosuppressive drugs within one month after receiving SOT to suppress subacute rejection. At this time, the immune system is greatly suppressed. However, this is also the high-risk period most likely to be infected by SARS-CoV-2, and once infected, it is also the easiest to develop severe illness [44]. A cohort study reported that among all kidney transplant recipients suffering from COVID-19, most of them underwent kidney transplantation within one year. This may mean that immunosuppression makes transplant patients more susceptible to SARS-CoV-2 infection [10]. Kidney transplant recipients who received immunosuppressive therapy have a higher probability of SARS-CoV-2 infection, duration of disease, and severity of symptoms than ordinary people with COVID-19 who did not receive any immunosuppressive therapy [45]. This result is similar to the conclusion of previous studies that the intensity of immunosuppressive therapy affects the recovery of infectious diseases (e.g., cytomegalovirus infection) in kidney transplant recipients [46]. However, the results from different research groups were not completely consistent on the same topic. In the inconsistent results, Hartzell et al. could not distinguish the differences in humoral response in SOT recipients with mild, moderate, or severe COVID-19. At the time of diagnosis of COVID-19, anti-proliferative agents do not seem to affect the antibody response [47]. The researchers found that impaired immunity in kidney transplant recipients does not affect antibody formation in COVID-19 [4]. In summary, the impact of SARS-CoV-2 infection on the immune system of SOT recipients and the impact of immunosuppression on the severity and progression of COVID-19 require more clinical evidence to clarify.

## 3. Use of Immunosuppressive Agents in Kidney Transplant Recipient with COVID-19

The immune response generated by the immune system for grafting is one of the main factors affecting graft survival. The recipient’s immune system treats the donor kidney as a foreign object and rejects it. Therefore, proper modulation of the immune system is very important for the survival of kidney transplant recipients and the useful life of the donor graft. The current clinical immunosuppressive therapy for kidney transplant recipients is primarily divided into two stages: initial immunosuppressive induction therapy and maintenance immunosuppressive therapy. Initial immunosuppressive induction therapy refers to strong and short-term immunosuppressive effects during the perioperative period and the immediate postoperative period. Maintenance immunosuppressive therapy is followed by initial immunosuppressive induction therapy to maintain moderate suppression of the immune system. The focus of this article is on the impact of the drugs used in maintenance immunosuppressive therapy on COVID-19 [2]. During maintenance immunosuppressive therapy, patients are given several kinds of drugs that target different immune responses and mechanisms in the immune system to suppress rejection of foreign organs. Currently, most organ transplant centers use triple-drug regimens to treat patients receiving SOT to suppress chronic rejection, including corticosteroids, calcineurin inhibitors (CNIs), and mycophenolic acids (MPAs) [2]. In addition, mammalian targets of rapamycin inhibitors (mTORi), azathioprine, IL-2 receptor antagonist, polyclonal antibodies, and monoclonal antibodies are immunosuppressive drugs that can be applied to SOT patients [2,48]. This article discusses corticosteroid, CNI, MPA, and mTORi in COVID-19.

### 3.1. Corticosteroids

Corticosteroids are widely used as immunosuppressants for patients receiving SOT. Corticosteroids affect the immune and inflammatory responses in patients receiving SOT by activating anti-inflammatory genes or suppressing the expression of inflammation-related genes. For example, it can reduce the release of cytokines, down-regulate pro-inflammatory mediators, stimulate the release of anti-inflammatory mediators, and suppress coagulation factors. In addition, corticosteroids affect the differentiation and ability to generate cytokines in monocytes, macrophages, and T-cells [49,50]. Glucocorticoids bind to the steroid receptor in the cell, and the glucocorticoid–steroid receptor complex translocates to the nucleus, thereby adjusting the expression of inflammatory genes. Glucocorticoids also inhibit the activation of transcriptional factors/modulators, such as NF-κB or AP-1, which are related to inflammation, and have anti-inflammatory and immunosuppressive effects [51,52]. Treatment with corticosteroids is of great importance for kidney transplant recipients. However, adjusting the use of corticosteroids for kidney transplant recipients with COVID-19 is also are search focus. In the past treatment of SARS and MERS, it was found that the use of corticosteroids increased the mortality rate and the risk of secondary infection and impaired the patient’s ability to remove viral particles as well as prolonged the viral shedding [53]. Systemic steroid treatment may not work for decreasing death, duration of hospitalization, as well as period of COVID-19 viral shedding [54]. Therefore, in the early stages of the COVID-19 pandemic, many clinicians believed that the use of corticosteroids should be restricted during the treatment of COVID-19. A physician used corticosteroid-sparing immunosuppression with dose reducing antiproliferative agent and tacrolimus on a kidney transplant patient suffering from COVID-19 to avoid graft rejection and found that renal transplant recipients with moderate oxygen requirements could achieve the purpose of immunosuppression without corticosteroid. Moreover, it will not cause the deterioration of COVID-19 disease and avoid the occurrence of graft rejection, let alone the side effects caused by corticosteroids. Therefore, it is believed that in the immunosuppression regiment of COVID-19 infected renal transplant recipients, abandoning corticosteroids and maintaining the application of CNI and MPA may have better results for the recovery of patients.

However, after the development of the COVID-19 pandemic in 2021, clinical experts have a new idea of using corticosteroids. In a randomized evaluation of COVID-19 therapy (RECOVERY) trial involving a large number of patients, the experimental group containing 2104 COVID-19 patients was administered oral or intravenous dexamethasone (6 mg once daily for 10 days), and the control group containing 4321 COVID-19 patients was given normal care. The results showed that 21.6% of the patients in the experimental group who received dexamethasone and 24.6% of the patients in the control group who received normal care died within 28 days (rate ratio, 0.83; 95% confidence interval [CI], 0.75 to 0.93; *p* < 0.001). This result shows that the use of corticosteroids can significantly reduce the mortality of patients with general COVID-19, particularlyin patients with moderate to severe COVID-19 (who receive mechanical ventilation) [55]. Because clinicians consider that corticosteroids have no negative effect on the treatment and course of COVID-19 at this time, it has a positive effect on continuous control of chronic rejection. Angelico et al. analyzed 554 SARS-CoV-2 patients who received kidney transplants andrevealed that approximately 72% of patients will have their clinicians decide to continue using corticosteroids as an immunosuppressive agentduring the treatment of COVID-19 [56]. Marik et al. lso supported the provision of corticosteroids for kidney transplant recipients suffering from COVID-19. They also proposed the MATH+ protocol, which includes methylprednisolone, ascorbic acid, thiamine, heparin, supplemental oxygen, and other antiviral treatments for COVID-19 patients [57]. Marik et al. considered that the dosage of dexamethasone (6–30 mg methylprednisolone equivalent) used in the aforementioned RECOVERY study was extremely low. Fora better curative effect, sufficient plasma corticosteroid concentration is required to achieve maximal saturation of the steroid receptor (approximately 80 mg of methylprednisolone equivalent) [58]. Additionally, a study has revealed that corticosteroid therapy has a negative effect on the asymptomatic or early symptomatic phase of COVID-19 patients (without the need for supplemental oxygen). Corticosteroids should only be used in patients with moderate-to-severe COVID-19 [58]. Rosa-Guerrero et al. described the success of the treatment in a case report. They used high-dose intravenous immunoglobulin (IVIG; 65 g/day) combined with steroid pulses (giving a high dose of steroid in a short time, 125 mg/day of 6-methylprednisolone) to treat SARS-CoV-2-infected kidney transplant recipients [59]. Such treatment can effectively remove inflammatory cytokines (ferritin, D-dimer, CRP, lactate dehydrogenase, and IL-6) in the plasma and maintain hemodynamic stabilization in a short period. Damage to kidney function can also recover quickly. More importantly, there is no allograft rejection or donor-specific anti-HLA antibody production after COVID-19 is cured. In short, continuous administration of corticosteroids to kidney transplant recipients suffering from COVID-19 can prevent allograft rejection and suppress the severe immune response caused by SARS-CoV-2, and thus, patients can have a better recovery [60,61].

Methylprednisolone is a corticosteroid drug that has the effect of optimal lung penetration and can regulate the expression of inflammatory genes. Clinicians speculate that it has a good effect on patients suffering from COVID-19; however, in fact, the treatment of methylprednisolone at different timings and dosages will result in a significantly different prognosis in COVID-19 patients. Clinical reports have demonstrated that COVID-19 patients treated with methylprednisolone have a higher morbidity of severe symptoms, which can easily develop into ARDS; however, methylprednisolone can also reduce the mortality of patients with ARDS [62]. Clinical experts believe that the use of high-concentration methylprednisolone (pulse therapy) for a short period of time, compared with continuous administration of corticosteroid, will not over-suppress the immune system’s response and will not affect the viral clearance and production of specific IGg, and can also inhibit the cytokine storm subsequently to improve the illness condition in patients. Additionally, the study also emphasized that the main efficacy of methylprednisolone lies in its anti-inflammatory effect; therefore, it needs to be used in severe COVID-19 or patients who have developed ARDS. If it is given in the early stage of the COVID-19 clinical course, it may result inpoor prognosisin patients [63,64]. Therefore, timely and appropriate application of methylprednisolone is very important in kidney transplant recipientssuffering from COVID-19 [62,65].

In short, when kidney transplant recipients have asymptomatic or mild COVID-19, corticosteroid treatmentas an immunosuppressant may have a negative impact on the patient’s recovery. However, when COVID-19 progresses to a severe situation, it is still recommended that corticosteroid treatment be continued, and the administration of short-term and high-dose corticosteroids will have a good therapeutic effect on severe COVID-19 and prevent graft rejection.

### 3.2. CNIs

CNIs are inexpensive and easily available immunosuppressants. It primarily includes three drugs: cyclosporine A (CyA), tacrolimus, and pimecrolimus. Cyclosporine binds to intracellular cyclophilin, and tacrolimus binds to tacrolimus binding protein. These complexes bind to cellular phosphatase calcineurin (CnA) and subsequently inhibit the function of CnA. When the CnA of T cells is inhibited, the dephosphorylation and activity of the nuclear factor in the cell will be in an inactive state, which in turn will reduce the expression of pro-inflammatory molecules such as TNF-α and IL-2 [66]. Clinically, treatment with these drugs is very important for the treatment of SOT recipients. It can suppress the immune response, thereby preventing graft rejection and prolonging the period of graft survival. Angelico et al. revealed that approximately 90.61% of kidney transplant recipients with COVID-19 received maintenance immunosuppression therapy with CNIs as the mainstay. When COVID-19 progresses to moderate to severe situations, 19.7% of the patients received a reduced the dosage of CNI, and 31.9% of the patients suspended the treatment of CNI [56]. Clinicians believe that CNIs have a less negative impact on the course of COVID-19. Therefore, for kidney transplant recipients suffering from symptomatic COVID-19, clinicians should first choose to reduce or stop ADs or mTOR inhibitors instead of CNIs, and when the illness progresses to severely symptomatic COVID-19 at that time, the withdrawal of CNIs will be considered [23,56]. The results of the TANGO International Transplant Consortium also showed that clinical experts preferred to continue to use CNIs as immunosuppressants instead of mycophenolate (MMF/MPA) or mTOR inhibitors.

There are still many different opinions about which CNI should be selected by SOT recipients, which has less negative impact and even beneficial effects on the course of COVID-19. Angelico et al. showed that most clinicians chose tacrolimus to treat kidney transplant recipients with symptomatic COVID-19; 81.76% of patients used tacrolimus, 5.05% used CyA, and 3.79% used non-specified CNI [56]. However, studies have reported that CyA inhibits the replication of several coronaviruses *in vitro*, and this effect is independent of its immunosuppressive effect at noncytotoxic concentrations [67,68]. It also has the effect of early blockage of viral RNA and protein synthesis [68,69]. Previous evidence has showna high redundancy of SARS-CoV interaction with cyclophilin, which is the upstream element of the CnA/NFAT pathway, in vitro. CyA can inhibit the replication of coronavirusby binding to cyclophilin in cells. SARS-CoV activates the NFAT pathway through its non-structural protein 1 (Nsp-1) to produce cytokinesand induce immune activation, and CyA can reduce virus-induced cytokine production by inhibiting the NFAT pathway [70]. Sauerhering *et al.* revealed that CyA combined with cyclophilin can inhibit the replication of MERS-CoV, reduce cell foci formation, and protect epithelial integrity in *in vitro* and animal experiments [71]. CyA also reduces the MERS-CoV titer in cells by inhibiting the activation of c-Jun N-terminal kinase (JNK) of mitogen-activated protein kinases (MAPKs).Interestingly, CyA stimulates epithelial cells in the lungs to produce a large amount of interferon regulatory factor 1 (IRF1), type III IFN (IFN-λ), and multiple interferon-stimulated gene (ISG) activation, which in turn produces a robust IFN-λ response and inhibits viral proliferation, significantly improving the severity of the disease. CyA also has a potential effect in the treatment of hemophagocytic lymphohistiocytosis (HLH) with cytokine-release syndrome. Researchers believe that CyA may improve the excessive release of pro-inflammatory cytokines caused by COVID-19. CyA has a curative effect on the hyper-inflammatory phase of severe COVID-19 [72]. Therefore, some experts suggest that replacing tacrolimus with immunosuppressive agents used by SOT recipients with symptomatic COVID-19 with CyA can effectively reduce the mortality of patients [22,73,74]. Although some clinicians do not recommend the use of tacrolimus for SOT recipients with symptomatic COVID-19, a study on liver transplant patients showed that tacrolimus has more protective potential than CyA, mTOR inhibitors, and MPA [75]. A previous case report also demonstrated that the use of tacrolimus by kidney transplant recipients has a positive effect on the course of COVID-19 [76]. Tacrolimus, like CyA, also inhibits human coronavirus replication by binding to cyclophilin in cells [77,78]. The study by Solanich et al. found that the treatment of severe COVID-19 patients with methylprednisolone pulses plus tacrolimus can improve clinical outcomes and reduce patient mortality [79]. There are still disagreements regarding the use of tacrolimus in SOT recipients suffering from COVID-19.

CNI and mTORi are primarily metabolized by cytochrome P450 (CYP3A4), CYP3A5, and efflux pump P-glycoprotein. They will interact with the antiviral drugs currently used in COVID-19, such as P-glycoprotein substrate, chloroquine and hydroxychloroquine or CYP3A4 substrate, and lopinavir/ritonavir to produce competitive inhibition. The drug interactions can lead to abnormal drug metabolism and high serum concentrations of immunosuppressants and antiviral drugs, which can lead to side effects, such as QT prolongation or drug toxicity. Therefore, in the treatment of transplant patients suffering from COVID-19, if the above-mentioned drugs are used, it is necessary to monitor whether the concentration of CNI in the blood meets the therapeutic range [38,80,81,82].

Regarding the use of CNIs in kidney transplant recipients with symptomatic COVID-19, clinical experts still have many different opinions. At present, clinical experts prefer to continue using CNIs under strict monitoring of blood concentration or dosage reduction. In short, there is still a need for more treatment experience to guide the adjustment of drug use.

### 3.3. Mycophenolic Acid (MPA)

Mycophenolic acid (MPA) is a selective, non-competitive, and reversible inhibitor of inosine-5′-monophosphate dehydrogenase (IMPDH) [83]. At present, the main drugs used in clinical practice are MPA prodrugs, such as mycophenolate mofetil (MMF) and salt mycophenolate sodium. MPA inhibits the proliferation of T and B lymphocytes and the production of immunoglobulins through the guanosine and deoxyguanosine nucleotides in depleted lymphocytes, thereby producing immunosuppressive effects [83]. Many *in vitro* and animal studies have pointed out that MPA can inhibit the replication of MERS-CoV [84,85,86] by inhibiting the papain-like protease activity of the virus [86,87]. A synergistic effect will occur when MPA is used with interferon [85,86], stimulating the expression of interferon-stimulated genes and causing the cell to enter the state of resisting virus replication [88]. However, many studies on MPA drugs that inhibit virus replication in the past excluded SARS-CoV and SARS-CoV-2. Studies have shown that MPA has no way to reduce the virus titer in the lungs when a mouse is infected with SARS-CoV, and it has more severe symptoms compared to a mouse that has not received MPA treatment [89]. *In vitro* experimental results have shown that MPA cannot inhibit the growth of SARS-CoV-2 [90]. Therefore, Angelico *et al.* emphasized in a systematic review that clinicians typically do not alter the original immunosuppressive use of asymptomatic or mild symptoms in kidney transplant patients after suffering from COVID-19. They typically use mycophenolic acid combined with CNI and corticosteroids to maintain the immunosuppressive effects in patients; however, when the patient’s course of COVID-19becomes symptomatic, most patients will be stopped from using MPA [56]. If the patient’s COVID-19 is mild to moderate or if they are a young patient without comorbidities, most doctors will withdraw MPA and maintain the use of other immunosuppressive agents. These results indicated that most clinicians conservatively use related drugs in the treatment of kidney transplant recipients with symptomatic COVID-19 due to the influence of MPA on the adaptive immune response and its nephrotoxicity [91]. Summarizing the current research results showed that the use of MPA in the treatment of kidney transplant recipients with symptomatic COVID-19 may not have sufficient positive effects. Therefore, it is recommended to reduce or stop the use of MPA for the prognosis of patients.

### 3.4. Mammalian Target of Rapamycin Inhibitor (mTORi)

Mammalian target of rapamycin inhibitors (mTORi) includes everolimus and sirolimus, which bind to the intracellular protein FKBP-12 and form a complex, thereby inhibiting the activation of mTOR complex-1 and 2. After the activation of the mTOR complex-1 and 2 is inhibited, the translation and synthesis of related genes will be inhibited, thereby inhibiting the proliferation of lymphocytes to achieve an immunosuppressive effect [92]. In the past, in an *in vitro* experiment, it was shown that everolimus may inhibit the mTOR signaling pathway and subsequently inhibit MERS-CoV replication [93]. However, another study demonstrated that mTORi weakens the function of IL-37, thus making IL-37 unable to effectively inhibit the production of IL-1 during SARS-CoV-2 infection, thereby making the downstream inflammatory response unable to be inhibited [94]. In addition, mTORi may cause drug-induced pneumonitis and interstitial lung disease, which have a high incidence rate in kidney transplant patients [95,96,97]. SOT recipients who receive mTORi as an immunosuppressant have a higher probability of producing proteinuria, which is a prognostic factor of kidney graft survival, than patients who receive CNI, and mTORi may also cause side effects such as anemia and venous thromboembolism in transplant patients [95,97]. Therefore, clinicians are hesitant to administer mTORi treatment to kidney transplant recipients with symptomatic COVID-19. According to statistics, approximately 8.66% of kidney transplant recipients with mild symptomatic COVID-19 will be given mTORi; when the patient’s symptoms aggravate from moderate to severe, approximately 79.2% of patients will be discontinued from the administration of mTORi [56]. From these data, it can be seen that the penetration rate of mTORi in transplant patients infected by SARS-CoV-2 is lower than that of other immunosuppressive agents, which also means that current clinicians believe that mTORi may provide more injuries in SOT recipients suffering from COVID-19, and there are other drugs that can replace it to provide anti-rejection effects. We have described how the antiviral agents used in the treatment of COVID-19 may inhibit the metabolism of mTORi and cause harmful side effects. Therefore, it is necessary to be more cautious and strictly monitor the patient’s drug plasma concentration when using mTORi [81,82]. In short, transplant patients suffering from COVID-19 should stop using mTORi and use CNI to maintain the immunosuppressive effect of the drug and reduce the dose of CNI or increase the interval between administration to reduce side effects [38,81].

## 4. Conclusions

According to the above discussion, the use of immunosuppressive agents is very important for the treatment of kidney transplant patients after COVID-19. Clinicians must also make appropriate adjustments according to the patient’s COVID-19 progress to avoid unnecessary side effects that may impair the recovery of COVID-19.Clinicians can choose to reduce or stop the use of corticosteroids after the patient is asymptomatically infected with COVID-19 or at the initial stage of symptomatic COVID-19.As CNIs are the key agents used to inhibit chronic rejection after patients receive SOT, CNIs and antiviral drugs such as chloroquine, hydroxychloroquine, and lopinavir/ritonavir are prone to drug–drug interaction. Therefore, the choice of CNIs must be considered and the dosage needs to be monitored via the serum concentration when administered to kidney transplant recipients with COVID-19. When the patient’s COVID-19 changes from asymptomatic to symptomatic or symptomatic COVID-19 changes from moderate to severe, clinicians can consider reducing the dosage or suspending the administration of CNIs, and switch to methylprednisolone pulse therapy to prevents systematic hyper-inflammatory syndrome and graft rejection. Although MPAs and mTOR have partial antiviral effects, the current research results do not recommend the use of these drugs for kidney transplant patients with COVID-19.

## Data Availability

Data sharing not applicable.
